# Mechanics of Uterine Displacements

**Published:** 1876-09

**Authors:** A. Howard Scott

**Affiliations:** Mansfield, Ill.


### MECHANICS OF UTERINE DISPLACEMENTS.

By A. HOWARD SCOTT, M. D., Mansfield, Ill.

(Read before the Central Illinois Medical Society, May 2, 1876.)

I shall endeavor first to give the dynamics of uterine displacements, tracing out separately the mechanism of each, so that we may obtain clearer views of the indications to be met by mechanical means in our management of them.

There are three necessary conditions to constitute a healthy action of the uterus: 1st, A normal circulation and innervation; 2d, a normal structure; 3d, a normal position. These inseparable conditions constitute a tri-link chain, absolutely necessary for the existence of its healthy action.

All uterine pathological processes must take their origin in one or the other of the links of this physiological chain. If it start in the first link with disordered circulation and innervation, this may lead to abnormal structure (hyperplasia), and this abnormal structure may eventuate in abnormal position. If the morbid process start in the second link, with subinvolution following parturition, this may affect either the first or the third link, in the one case giving hyperplasia, in the other displacement. And finally, if the pathological process take its origin in the third link, with displacement resulting from any of the various forces usually producing it, this must of necessity soon affect the circulation and innervation, which later may eventuate in change of structure.

Before discussing the dynamics of displacements, we must briefly examine uterine statics, or the physiological forces in a state of equilibrium.

If we remember that the inherent weight of the uterus and superadded weight of abdominal vicera, constitute a force tending to carry the uterus lower in the pelvis, and that the vagina and the various ligamentous supports constitute opposing forces, and that when there is an equilibrium between these forces the uterus is in its normal position, we may express the statical condition by the following equation: Weight of uterus + weight abdominal vicera = resistance of vagina + resistance of ligaments. Now it is evident that any change of the one side of this equation, without a corresponding change of the other, must destroy its equality, giving as a result either an ascent or descent, or an anterior or posterior displacement of the uterus. Those proximate causes and favoring conditions usually enumerated by authors in their etiological classifications, we shall consider as so many physico-pathological forces, and arrange them thus:

*Direct Forces.*—Subinvolution, hyperplasia, uterine tumors, fluids contained in uterus, pregnancy, abdominal tumors, ascites, abscess, hæmatocele, distended bladder, distended rectum, tight lacing, heavy skirts, muscular efforts.

*Indirect Forces or Diminished Resistance.*—Ruptured perineum, weakened vaginal wall and sphincter, weakened uterine ligaments, weakened levator ani and perinei muscles, tissue change of cervix.

To properly correlate these various forces and to trace out their reciprocal actions and relations in the successive pathological and physical changes, constitutes one of the most complex problems in the science of etiology, we can best approximate the solution of this problem by studying in detail these various displacements. We shall therefore consider only the more important of them, in the following order: 1st, Prolapsus; 2d, Versions; 3d, Flexions.

Before proceeding further it becomes necessary at least to enumerate the various uterine supports, which we will divide into two classes: 1st, Those connected with the cervix; 2d, Those connected with the body.

In the first class we enumerate the utero-sacral and the utero-vesical ligaments, together with the vagina, the most important and unyielding of all the supports, being so intimately connected by areolar tissue to the rectum and bladder as to involve these viscera in its descent. They act as guys to the cervix as well as support to the corpus. In the second class we enumerate the broad and the round ligaments, together with the bladder, as guys only to the body of the uterus, they offering no resistance to the descent of the uterus until procedentia is attained.

So delicately is the uterus poised in the pelvic cavity that each act of inspiration give a transitory physiological prolapsus. Need we then be surprised that a pathological prolapsus should be of such frequent occurrence? That physiological prolapsus peculiar to the first few months of utero-gestation, affords the best type by which to study the mechanism of the displacement. Here we have the increased weight of the uterus acting as a vertical force, together with the supports physiologically relaxed, combining to bring about the given result, prolapsus. This teaches that it requires little or no additional force to be added to the normal weight of the uterus and abdominal viscera to bring about prolapsus if the supports be enfeebled. Then it matters not what that superadded force may be, whether fœtus, tumor, hyperplasia, subinvolution, ascites, tight lacing or heavy skirts, the results will be the same if the supports be weakened; nay, prolapsus may occur in spite of the normal resistance of supports.

Then we may formulate the following as the mechanism of prolapsus: A force or combination of forces acting with or upon the uterus vertically, forcing it lower in the pelvis — this force or combination of forces being greater than the combined resistance of vagina, utero-sacral and the utero-vesical ligaments, together with the resistance of the round and broad ligaments when the descent amounts to procedintia. So that we have as a physico-pathological result increased length of all of the ligaments, the vagina being prolapsed or shortened.

We may express this displacement by the following formula, x denoting the super-added direct force, whatever it may be, thus: Weight uterus + weight abdominal viscera + x = resistance vagina + resistance of all the ligaments.

In retro-version the bladder and rectum play important parts. A distended rectum thrusts the cervix forward, putting the utero-sacral ligaments on the stretch, and if a replete bladder has already elongated the broad ligaments, we have a physical condition of the proper ligaments that will give a retroversion, if the same direct forces be in operation as in prolapsus. Then we formulate for retroversion the following: A combination of vertical force or forces with a horizontal force or forces, acting jointly with or upon the uterus, giving a resultant oblique force which carries the fundus backwards and downwards, dislocating cervix and top of vagina forward, this complex force or forces being greater than the resistance of round and utero-sacral ligaments, which we may likewise express by a formula, thus: Weight of uterus + weight of abdominal viscera + distended rectum + distended bladder + x = resistance of round ligaments + resistance of utero-sacral ligaments. Observing similar ætiological conditions, we may express the equation of *ante*-version thus: *Weight of uterus + weight viscera + x = resistance of utero-vesical ligaments.*

Flexions, as a rule, are the sequelæ of versions; this I have frequently seen demonstrated. The uterus in retroversion lies across the recto-vaginal septum, and in ante-version it lies across the vesico-vaginal septum. This change of position soon induces a change of circulation and innervation, which eventuates in tissue change of the organ, rendering it soft and spongy. The organ being in this condition it requires no effort of the imagination to see that if the same vertical forces be acting on the organ, they will still further depress the fundus and cervix, thus bending it across the septum, giving a retro-flexed or ante-flexed uterus, as the case may be. Authors usually claim cervical tissue change as the determining cause of flexions. The organ being soft and spongy from the nature of this change, the same forces that would tend to give a version, would give a flexion if the cervical support be firm.

Then for flexions we have the same formula as for versions, except that in flexions the resistance of the cervix is less than the resistance of the cervical ligaments. For retro-flexion we express it thus: Displacing force > resistance of cervix + resistance of round ligaments. For ante-flexion: Displacing force > resistance of cervix.

Then, to recapitulate, we will group together these formulas, in order to show at a glance the supports at fault in these respective displacements. For brevity we use the initials only of previous formulas:

*Normal Uterus.*—Wt. U. + Wt. A. V. = resistance vagina + resistance of all ligamentous supports. (x = cause.)

*Prolapsus.*—Wt. U. + Wt. A. V. + x > resistance vagina + resistance of all ligaments.

*Retroversion.*— Wt. U. + Wt. A. V. + distended bladder + distended rectum + x > resistance round ligaments + resistance utero-sacral ligaments.

*Ante-version.*—Wt. U. + Wt. A. V. + x > resistance of utero vesical ligaments.

*Retro-flexion.*—Wt. U. + Wt. A. V. + x > resistance round ligaments + resistance of cervix.

*Ante-flexion.*—Wt. U. + Wt. A. V.—x > resistance of cervix.

These formulæ give us clear views of the ligaments at fault, and thus point out the indications to be met in the employment of mechanical supports. Before considering these supports, I will make a few remarks on the general management of these cases. The first indication to be met, will be to remove those forces and determining causes that we have enumerated in our etiological classification. The second indication will be to restore the dislocated uterus to its normal position, and retain it there by appropriate mechanical contrivances until the uterine supports regain their normal length and tone. These second indication we shall only consider in this article. The means of reducing a displaced uterus are so well understood that I shall not consider them, but at once proceed to a consideration of mechanical supports.

*Pessaries* have been used from time immemorial. Their almost innumerable varieties teach plainly the unsatisfactory nature of all such supports as have hitherto been devised. Though this is in a great measure owing to the employment of pessaries before the pathological conditions associated with the displacement have been removed by appropriate means, yet much of the difficulty has been, I apprehend, in not having properly constructed instruments—instruments having an eye to the supports at fault and to an objective cure. The "coming pessary" must have a regard for these things — one that will not distend the vagina nor chafe, and will at the same time retain the uterus in its normal position.

The most philosophical instrument yet devised for prolapsus uteri is, undoubtedly, the stem surmounted with a cup to receive the cervix. It fills the indications with the least distension of vagina; but it is objectionable, practically, because the patient is liable to place the cup in the anterior or posterior cul-de-sac, as over the cervix, and may thus do themselves incalculable injury and unjustly blame the instrument for it. On this account a stem surmounted by a globe or pear shaped body would be safer in the hands of the majority of women, and generally give good results. All stem pessaries, however, are liable to chafe the posterior fourchette and the labia majora; yet by giving a proper curve to the stem, and a little patience on the part of wearer, this objection may in time be overcome. But if a stem instrument cannot be tolerated, then employ Hodge's closed lever, the elastic ring or hard rubber disk-pessary. The latter is an instrument generally tolerated well by the vagina, and usually gives good results in this displacement, yet it is objectionable in distending the vagina too much. We should have an eye to the vagina regaining its normal calibre, as well as the ligaments regaining their normal length, in our treatment of all displacements, but of this one especially.

In retroversion the round and utero-sacral ligaments are chiefly at fault, both being elongated. The indications here are to carry the cervix upwards and backwards, throwing the fundus forward, and to keep them thus until the utero-sacral and the round ligaments regain their normal tone and length. The most philosophical instrument for this purpose, that I

have yet seen, is the pessary of Schultze, that I found described by Schroeder in his treatise on diseases of the female sexual organs. The short curve of this instrument presses against the anterior face of the cervix and keeps it well back, without putting the utero-sacral ligaments to further stretching, a thing we should avoid. This instrument, modified by a sub-pubic curve, is much improved. Should this intrument not be tolerated, I should then employ a stem surmounted by a globe or pear shaped body, adjusted in front of cervix (not the corpus). Those so-called retroversion pessaries, that carry the cervix backward by stretching the posterior vaginal wall, and exert a leverage action through the fornix vagina, I consider unphilosophical, if not injurious. The facts in this displacement are, that the posterior vaginal wall and utero-sacral ligaments have already been stretched to their utmost in the forward movement of the cervix. The surgeon, in his efforts to radically cure this displacement, shortens the posterior vaginal wall, the very opposite effect to that accomplished by one of the so-called retroversion pessaries.

In ante-version the utero-sacral and round ligaments both become shortened as a result of this displacement. The indication then is to lengthen them both by carrying the cervix forwards and the fundus upwards and backwards. There is no means yet devised to meet the indications philosophically, without putting the already elongated utero-vesical ligaments to a further stretching, yet we are justified in doing so in order to effect an elongation of the round ligaments. Then the most appropriate instrument to effect this, I think, is Thomas' modification of the Cutter pessary. It has this advantage over Thomas' ante-version pessary, the patient can adjust and remove it herself. After having worn it sufficiently long to effect an elongation of the round ligament, then use the stem and ball pessary, adjusted behind the cervix, so as to throw it forward and give the utero-vesical ligaments opportunity to contract, at the same time employing a truss-like pad, pressing the hypogastrium just above the symphisis pubis, to prevent any forward inclination of the uterus. This procedure usually gives gratifying results.

In flexions the round ligaments are chiefly at fault (all the other ligaments being normal, or nearly so), they being elongated in retro-flexion and shortened in ante-flexion. The indications in the one case being to carry the fundus upwards and forwards, in the other to carry it upwards and backwards, and so retain it, if we can, until the round ligaments regain their natural length and tone. In flexions the so-called retroversion and ante-version pessaries are more appropriate and philosophical than they are in those displacements that their respective names indicate; but they all are palliative, not curative. We must first try to effect a straightening of the flexion; this we can often accomplish by a judicious employment of the sponge tent, after the plan so well described in the January number of the *American Journal of Medical Science*, by Dr. Ellerslie Wallace. I employed the sponge tent for this purpose ten years ago, and succeeded in relieving permanently one of the worst cases of retro-flexion I ever saw, accompanied with the most agonizing obstructive dysmenorrhœa. After having straightened the flexion, whilst the cervix is yet soft and spongy, employ any of the various ante-version and retroversion pessaries usually employed to elevate the fundus, until the round ligaments regain their lost tone and length. I have had no experience with the intra-uterine stem pessary for straightening flexions, as described and advocated by Schroeder, in his treatise on female sexual organs; yet I believe it to be one of the very best of instruments for a limited class of cases, those having no inflammatory complications.

For those cases of flexion with a long cylindrical cervix, the pessary invented by Dr. Hurd, of Alabama, is highly commended by gynarcologists as giving good results. I have never as yet employed it. I regret that time will not allow me to discuss further these flexions: to do them justice a separate monagraph should be written.

No matter what instruments we may employ in these various displacements, we should keep a close supervision over them, lest by pressure or chafing they do serious mischief. The cold vaginal douche should be employed freely morning and evening so long as the instrument is worn. Should at any time the uterus or vagina become intolerant of the instrument, remove it and employ tampons medicated with carbolic acid and glycerine, after the plan of Dr. T. G. Thomas, N. Y., when, after a few days, the instrument may again be resumed.

---

Impression produced on the Skin by Different Heated Liquids.—(Bloch. *Révue Medico-Photographique. Times and Gaz.*, 1876.) M. Bloch has communicated to the Société de Biologie the results of his experiments, which show that liquids become burning at very different temperatures. He takes as a term of comparison the thermometrical degree which allows of the immersion of the hand during two minutes without producing great pain. He finds that this is 48° C. for mercury, 49° for water and for solution of tannin, 51° for solution of carbonate of soda, 52° for alcohol and for milk, 55° for essence of turpentine and starch-paste, 57° for glycerine, 60° for oil, and 65° for beef-fat. He explains most of the differences by the greater or less facility with which imbibition by the epidermis takes place, and especially those observed in aqueous solutions and fatty bodies. This hypothesis cannot apply to mercury, and its action is probably explained either by its great conductibility, or by the pressure in vi rtue of its density which it exerts on the immersed part—a pressure which renders the contact closer and the propagation of caloric easier.

